# A Single Session of rTMS Enhances Small-Worldness in Writer’s Cramp: Evidence from Simultaneous EEG-fMRI Multi-Modal Brain Graph

**DOI:** 10.3389/fnhum.2017.00443

**Published:** 2017-09-05

**Authors:** Rose D. Bharath, Rajanikant Panda, Venkateswara Reddy Reddam, M. V. Bhaskar, Suril Gohel, Sujas Bhardwaj, Arvind Prajapati, Pramod Kumar Pal

**Affiliations:** ^1^Department of Neuroimaging and Interventional Radiology, National Institute of Mental Health and Neurosciences (NIMHANS) Bangalore, India; ^2^Cognitive Neuroscience Centre, National Institute of Mental Health and Neurosciences (NIMHANS) Bangalore, India; ^3^Department of Biomedical Engineering, New Jersey Institute of Technology Newark, NJ, United States; ^4^Department of Neurology, National Institute of Mental Health and Neurosciences (NIMHANS) Bangalore, India

**Keywords:** multi-modal graph theory analysis, simultaneous EEG-fMRI, Writer’s cramp, repetitive transcranial magnetic stimulation

## Abstract

**Background and Purpose**: Repetitive transcranial magnetic stimulation (rTMS) induces widespread changes in brain connectivity. As the network topology differences induced by a single session of rTMS are less known we undertook this study to ascertain whether the network alterations had a small-world morphology using multi-modal graph theory analysis of simultaneous EEG-fMRI.

**Method**: Simultaneous EEG-fMRI was acquired in duplicate before (R1) and after (R2) a single session of rTMS in 14 patients with Writer’s Cramp (WC). Whole brain neuronal and hemodynamic network connectivity were explored using the graph theory measures and clustering coefficient, path length and small-world index were calculated for EEG and resting state fMRI (rsfMRI). Multi-modal graph theory analysis was used to evaluate the correlation of EEG and fMRI clustering coefficients.

**Result**: A single session of rTMS was found to increase the clustering coefficient and small-worldness significantly in both EEG and fMRI (*p* < 0.05). Multi-modal graph theory analysis revealed significant modulations in the fronto-parietal regions immediately after rTMS. The rsfMRI revealed additional modulations in several deep brain regions including cerebellum, insula and medial frontal lobe.

**Conclusion**: Multi-modal graph theory analysis of simultaneous EEG-fMRI can supplement motor physiology methods in understanding the neurobiology of rTMS *in vivo*. Coinciding evidence from EEG and rsfMRI reports small-world morphology for the acute phase network hyper-connectivity indicating changes ensuing low-frequency rTMS is probably not “noise”.

## Introduction

Repetitive transcranial magnetic stimulation (rTMS) influences brain functional organization as well as task performance probably by modulating network connectivity beyond the stimulation period and zone (Yoo et al., 2008; Park et al., 2014) and has found a therapeutic role in several neurodegenerative diseases (Chou et al., 2015). Though rTMS has been used in a variety of disease conditions like migraine, stroke, depression, dystonia, pain syndromes etc. (Chervyakov et al., 2015), the mechanism of action of rTMS still eludes neuroscientists. Based on the evidence of the long-lasting clinical benefits following rTMS, several biological studies have proposed rTMS induced alterations to occur at neuronal, synaptic and genetic levels (Okano and Ohkubo, 2003; McKay et al., 2007; Pazur et al., 2007) by mechanisms like long-term potentiation (LTP) and long-term depression (LTD; Chervyakov et al., 2015). LTP increases synaptic strength, lasts longer and is associated with high-frequency stimulation (>5 Hz) and LTD results in LTD of synaptic strength and is often associated with low-frequency stimulation (<1 Hz; Duffau, 2006). Molecular mechanisms associated with LTP have delineated alterations in postsynaptic NMDA receptors showing an immediate effect lasting for 30–60 min and a long-term effect lasting for several hours, days or weeks (Pfeiffer and Huber, 2006; Sutton and Schuman, 2006; Chervyakov et al., 2015). Clinical studies have found modulations induced by rTMS to be site specific with improvement in clinical scores in Writer’s cramp (WC; Murase et al., 2005) and primary cervical dystonia (Pirio Richardson et al., 2015) associated with low-frequency rTMS over the primary motor cortex and not over supplementary motor cortex. Molecular imaging with PET using [11C] FLB 457 has reported modulations induced by rTMS to be disease specific as it has found increased dopamine release in the orbitofrontal cortices in Parkinson’s disease (Cho and Strafella, 2009) and reduced dopamine production in basal ganglia of healthy controls (HCs; Ko et al., 2008), following high-frequency stimulation of the left dorso-lateral prefrontal cortex. As most of the motor physiology studies have focused on the clinical benefits of rTMS, studies on the mechanism of action of rTMS are few (Thut and Miniussi, 2009) but, available studies have documented topographically specific changes in the fronto-centro-parietal leads within few minutes after 1 Hz rTMS in HCs (Brignani et al., 2008).

Neuroimaging has supplemented the understanding of short and long-term structural and functional changes following rTMS and has unraveled widespread alterations especially involving cerebellum and basal ganglia circuits. These regions were traditionally obscured in motor physiology methods due to limited spatial resolution. Functional imaging studies based on blood oxygen level dependent (BOLD) changes during task have revealed sensori-motor network reconfiguration (Baudewig et al., 2001; Yoo et al., 2008; Eldaief et al., 2011) lasting for up to 2 h after a single session of rTMS in HCs (Pleger et al., 2006). Enhancement of resting state cerebello-thalamo-cortical network connectivity in WC (Bharath et al., 2015) and essential tremor (ET; Popa et al., 2013) was also described. Therapy with low-frequency rTMS has reported changes even in non-motor networks like default mode network (DMN; van der Werf et al., 2010). Majority of the prior studies using resting state fMRI (rsfMRI) have used hypothesis-driven seed to voxel or Region Of Interest (ROI) to ROI-based analysis (Eldaief et al., 2011; Popa et al., 2013; Bharath et al., 2015), and have tested specific networks known to be associated with genesis or modulation of tremor. In contrast, data-driven techniques like graph theory analysis and independent component (IC) analysis measure whole-brain connectivity changes when a definitive hypothesis is unknown. Another important feature of data-driven techniques is that it can be used to seamlessly combine multi-modality data like fMRI and EEG (Yu et al., 2016). Graph theory analysis of rsfMRI assumes that human brain has a “Small-world” topology capable of optimally balancing local processing and global integration and achieves higher information transfer with low energy consumption (Watts and Strogatz, 1998; Rubinov and Sporns, 2010). It has been found that this small-world architecture of the human brain is altered in disease (Bullmore and Sporns, 2009; Ye et al., 2015; Bharath et al., 2016).

Though graph theory metrics are powerful to characterize human brain networks, their application in movement disorders have been sparse. To ascertain the usefulness of multi-modal graph analysis of simultaneous EEG-fMRI in understanding the neurobiology of a single session of low-frequency rTMS, we undertook this study in patients with WC. Our hypothesis was that rTMS will alter disease induced loss of small-worldness on EEG and fMRI based on the evidence of consistent short-term changes in experimental and motor physiology studies.

## Materials and Methods

### Participants

Eighteen patients with WC (mean age ± SD 37.25 ± 13.76 years) participated in this study after providing written informed consent. The study was approved by the Institute (NIMHANS) ethics committee for humans. All patients were evaluated in detail by a single movement disorder specialist (PKP). All patients were right-handed and medications were withheld for 1 week prior to the study. All subjects provided written informed consent. Patients with a structural lesion on MRI, prior brain, spinal or peripheral nerve trauma/surgery, claustrophobia and on neuroleptic drugs were excluded from the study. Secondary causes of dystonia were ruled out in all patients by appropriate investigations. None of the patients had botulinum toxin therapy during their lifetime and did not have dystonia at rest. Twenty age, gender, and education-matched HCs with no neurological or psychiatric illnesses from the HC imaging database formed the control group.

### Simultaneous EEG-fMRI Protocol and Experiment Design

All subjects underwent the simultaneous EEG-fMRI in eyes closed, awake and relaxed conditions for the entire 9.24 min data acquisition protocol. All patients underwent EEG-fMRI in duplicate, one prior to the rTMS (R1) and second immediately after the rTMS (R2). The average time delay between rTMS and R2 was 10 min. HCs were imaged only once and did not undergo rTMS as consent could not be obtained.

## Data Acquisition

The acquisition parameters were identical for R1, R2 in WC patients and for R1 in HC.

### EEG Data Acquisition

EEG data was acquired simultaneously with rsfMRI using 32-channel MR-compatible EEG system (Brain Products GmbH, Gilching, Germany) in a 3T scanner (Skyra; Siemens, Erlangen, Germany). The EEG cap (BrainCap MR, Brain Products) had 31 scalp electrodes placed according to the international 10-20 system electrode placement and one additional electrode for ECG. Data was recorded relative to an FCz reference and an AFz ground electrode (Gnd) using the Brain Recorder software (Version 1.03, Brain Products). Data was sampled at 5000 Hz to suppress MRI gradient artifact. The impedance between electrodes and scalp was kept below 5 kΩ. To prevent head movement, sufficient padding was used and ear plugs were provided to all subjects.

### rsfMRI Data Acquisition

Whole brain T2* weighted images were acquired using a spin echo sequence (TR = 3000 ms; TE = 35 ms; refocusing pulse 90°; 36 slices; 4.0 mm slice thickness in an inter-leaved manner with an FOV of 192 × 192 mm, matrix 64 × 64 voxels with no gap, matrix, voxel size 3 × 3 × 4 mm). A three-dimensional magnetization-prepared rapid acquisition gradient echo (MPRAGE) sequence was acquired (voxel 1 × 1 × 1 mm) for spatial registration and segmentation.

### rTMS Parameters

After R1, subjects were moved to another room adjacent to MRI with the EEG cap in place and rTMS was delivered using a Magstim Super Rapid stimulator (Magstim Co. Ltd, Whitland, UK) with a figure-of-eight coil configuration. rTMS was applied tangentially to the scalp with the handle pointing backward and laterally at an approximate angle of 45° to the mid-sagittal line, perpendicular to the presumed direction of the central sulcus. rTMS was given over the left premotor cortex (PMC) by delivering 900 stimuli (90% of resting motor threshold (RMT)) at 1 Hz for 15 min. The RMT was determined as the lowest intensity that produced motor evoked potentials of >50 μV in at least five out of 10 single-pulse TMS stimulation using a TMS stimulator. The TMS stimulator was attached to an electromyography machine from the first dorsal interosseous muscle using Ag-AgCl surface electrodes placed over the muscle in a belly-tendon arrangement.

## Data Analysis

**Figure 1:**
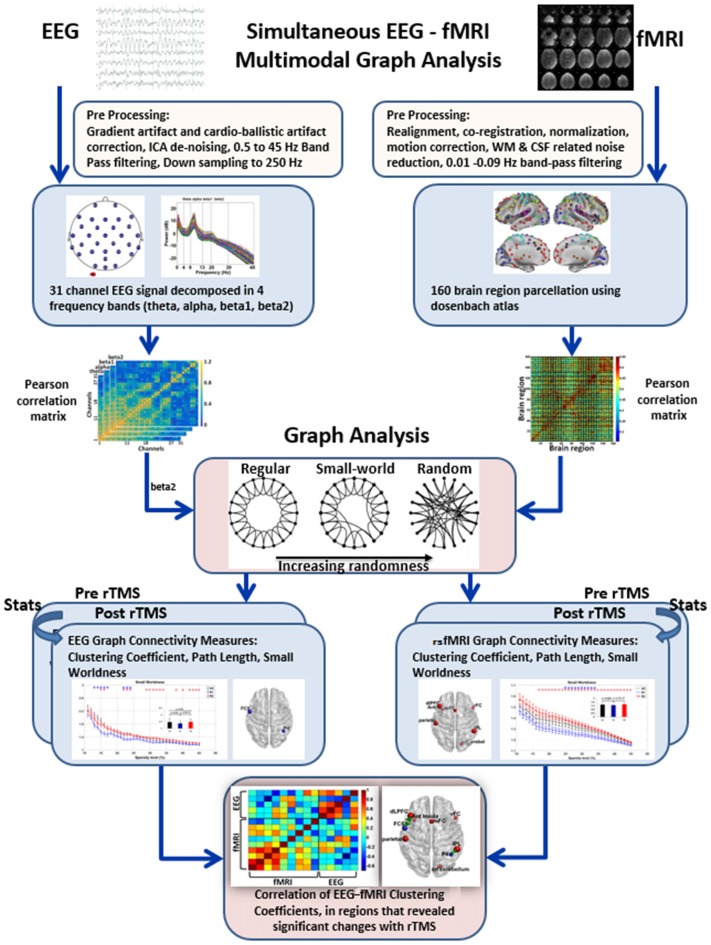
Overview of steps in multi-modal graph analysis of simultaneous EEG-fMRI. EEG and resting state fMRI (rsfMRI) were preprocessed in their respective toolboxes. Subsequently, Pearson correlation based connectivity in EEG and rsfMRI were used to derive graph metrics. Thereafter, paired *t* test was used between R1 and R2 to identify significant rTMS induced changes in the graph metrics in both EEG and rsfMRI. Finally, for multimodal EEG-fMRI analysis, simple correlation of the clustering coefficient in the regions that revealed significant changes in EEG and rsfMRI were obtained.

For both EEG and rsfMRI, preprocessing was done separately using their respective tools. The EEG and fMRI data were recorded for 185 dynamics (total 9.24 min), however, the first five dynamics (15 s/0.24 min) were excluded from both EEG and fMRI before preprocessing to avoid signal inhomogeneity during scanner start transition period. Subsequently, network analysis for both EEG and rsfMRI was carried out using the Brain Connectivity Toolbox^1^ (Rubinov and Sporns, 2010). Finally, correlation of the EEG and rsfMRI clustering coefficients was done. The overview of the steps is provided in Figure 1.

### Preprocessing

#### EEG

EEG data was preprocessed offline using BrainVision Analyzer software version 2 (Brain Products GmbH, Gilching, Germany; Sandhya et al., 2014) and scanner gradient artifact correction was done according to Allen et al. (2000) using a moving average width of 20 MR volumes (TRs). Ballistocardiogram (BCG) artifacts were removed by average subtraction method using heartbeat events (Allen et al., 1998, 2000; Goldman et al., 2000), implemented in BrainVision Analyzer 2. After removal of gradient and BCG artifacts, the data was down-sampled to 250 Hz. Muscular sources or head movement artifact and segments containing any channel variation more than 150 μV were removed visually. ICs were calculated using Independent Component Analysis (ICA) with the total number of ICA decomposition equal to the number of channels (i.e., 31). ICs which showed noise characters in temporal domain and spatial distribution were excluded through ICA back projection. Subsequently, the signal was filtered with a band-pass of 0.5–45 Hz. After offline preprocessing, EEG of each subject was visually inspected by an experienced researcher (RP) and the entire resting EEG was divided into 3-s epochs (180 epochs), each corresponding to simultaneously recorded rsfMRI. Epochs which contained obvious noise were rejected and epoch concatenation was performed. The EEG signal was subdivided into frequency bands, theta (4–8 Hz), alpha (8–13 Hz), beta1 (13–20 Hz) and beta2 (20–30 Hz) using the EEGLAB toolbox^2^.

#### rsfMRI

rsfMRI data preprocessing steps included realignment, segmentation of the structural data for regressing out the white matter and cerebrospinal fluid effects, normalization to MNI152 standard space of 3 × 3 × 3 mm^3^, motion correction using Friston’s 24-motion parameter, and temporal band-pass filtering with 0.01–0.09 Hz (Bharath et al., 2015). Data from four WC patients were removed due to higher head motion either in R1 or R2. Hence only 14 patients were included in the final analysis in both EEG and rsfMRI. The head motion was not significantly different between R1 and R2 (Translation (mean ± SD in mm):: R1: 1.05 ± 0.42, R2: 1.01 ± 0.43, p: 0.82; Rotation (mean ± SD in radians):: R1: 0.013 ± 0.005, R2: 0.016 ± 0.009, p: 0.19) using the Artifact Detection Toolbox (ART).

#### Brain Region Parcellation

In EEG, channels (*N* = 31) were taken as nodes of the functional networks and in rsfMRI, Dosenbach’s template was used to parcellate the brain into 160 functionally segregated ROIs (radius = 5 mm; Dosenbach et al., 2010) using MarsBaR toolbox^®^^3^. The rsfMRI time series of an ROI was defined as the average of all the voxels in that ROI.

## Graph Theory Analysis

Normalized clustering coefficient (*γ*), normalized path length (*λ*) and small-worldness (*σ*) were derived over the range of sparsity threshold to ensure the same number of network edges for each participant by retaining only those connections whose edge strengths exceeded a given threshold. By this procedure, the range of sparsity (0.11 ≤ *S* ≤ 0.45, with an increment of 0.01) was generated. This procedure guaranteed that the thresholded networks were estimable for small-worldness and also avoided excess network fragmentation at sparser thresholds (Fornito et al., 2010; Bharath et al., 2016).

For EEG, graph measures were calculated based on the N*N Pearson correlation connectivity matrix (*N* = 31 nodes) in four frequency bands as described above. For rsfMRI graph measures, time series was correlated region by region using Pearson’s correlation and a 160*160 matrix was constructed.

**Figure 2:**
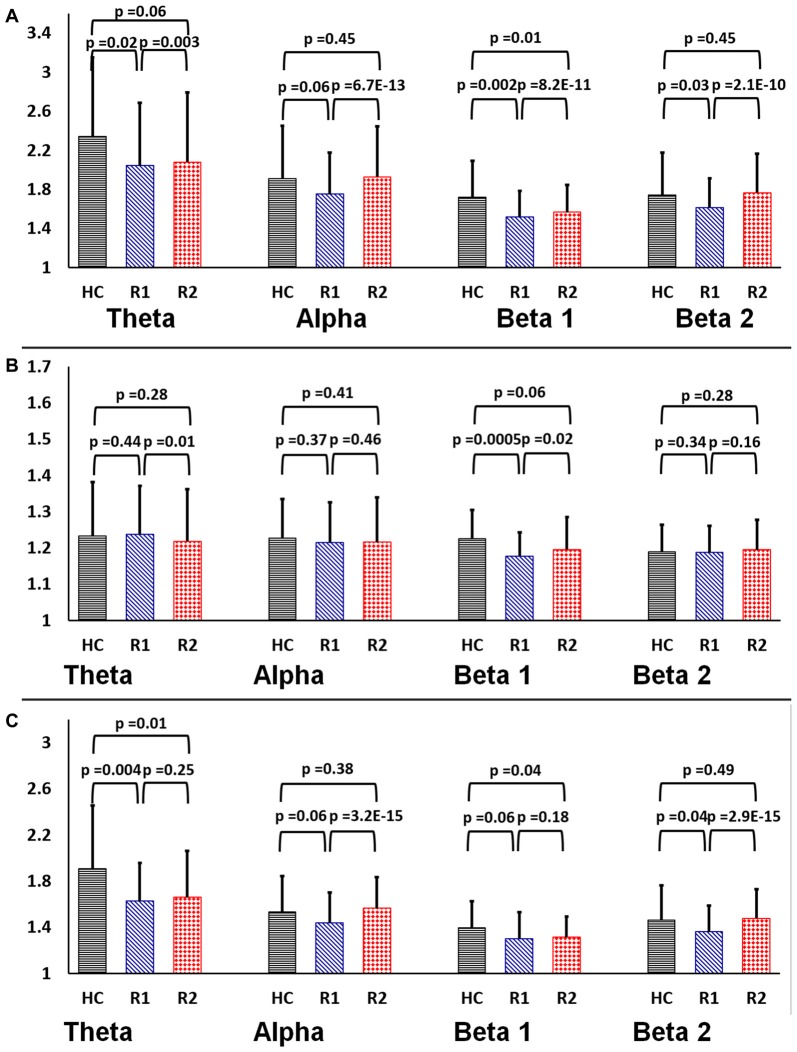
Rectangular bar graphs demonstrating group differences (healthy control (HC), R1 and R2) of mean **(A)** clustering coefficient **(B)** path length and **(C)** small-worldness across the four frequency bands in EEG.

The graph theory properties of the functional brain networks were defined on the basis of 31*31 in EEG and 160*160 Graph in rsfMRI, G (V, E) where G is the non-zero subset with vertices V = anatomical ROIs (nodes “N”) and edges E = Internodal correlation coefficient (Fisher’s *Z* value) as a connection between nodes were calculated. The small-world parameters (i.e., *γ*, *λ*, *σ*) were calculated over the range of sparsity (0.11 ≤ *S* ≤ 0.45) using the BCT toolbox^4^ and in-house MATLAB scripts (Fornito et al., 2010; Rubinov and Sporns, 2010; Bharath et al., 2016). Figure 2 demonstrates the distribution plot of *γ*, *λ*, *σ* in each of the frequency bands for HC, R1 and R2 groups in EEG. To limit the number of comparisons we have only used beta2 frequency band (20–30 Hz) for subsequent analysis as it showed significant differences in small-worldness between both HC-R1 and R1-R2.

## Statistical Analysis

Statistical analysis to derive group differences between HC, R1 and R2 was done for EEG and rsfMRI, separately. Two sample equal variance *t*-test with 5% of significance level was used between HC, R1 and HC, R2 comparisons and paired two-sample *t*-test was used for R1, R2 groups using the appropriate function in MATLAB 2013^®^. A false discovery rate corrected *p*-value of < 0.0062 for rsfMRI and 0.004 for EEG were taken as significant. Brain regions with significant differences in *γ* were used to identify associated networks in the Dosenbach’s atlas.

To determine if the rTMS induced changes in clustering coefficient in EEG was related to similar changes in the rsfMRI, the linear correlation of clustering coefficient between the EEG electrodes and rsfMRI brain regions were calculated across subjects. Regions showing significant rTMS induced *γ* changes in EEG and rsfMRI were furnished on a brain surface model using the Brain Net Viewer software (Xia et al., 2013).

## Results

**Figure 3:**
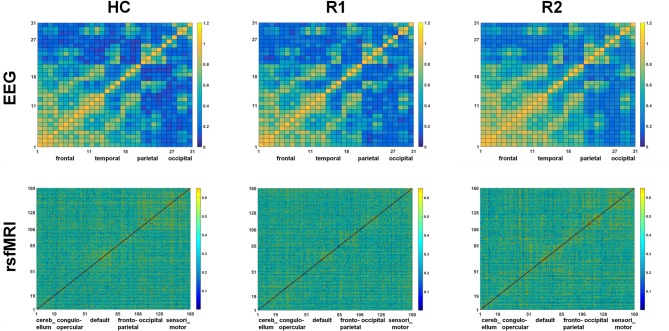
Comparison of the correlation metrics of 31 electrodes in the beta2 frequency range in EEG and correlation metrics of 160 regions in rsfMRI in HC, R1 and R2.

A single session of rTMS was found to significantly alter the network topology in both EEG and rsfMRI, with evidence of increasing *γ* and *σ* in several fronto-parietal areas. The correlation matrices of both EEG and rsfMRI in HC, R1 and R2 are provided in Figure 3.

### Changes in Network Topology

#### Evidence from EEG

**Figure 4:**
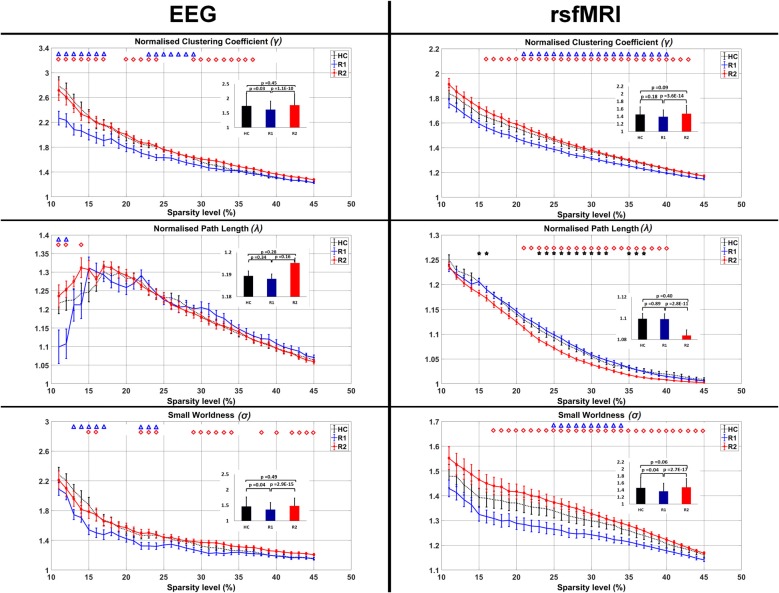
Comparison of the average clustering coefficient, pathlength and small-worldness in EEG and rsfMRI. The bar graph diagram in the inset shows the significance and the standard deviation. The blue triangle indicates the sparsity ranges that revealed significant differences between HC and R1, red quadrangle differences between R1 and R2 and black stars differences between HC and R2.

On EEG measures R1 showed significant reduction in *γ* (Sparsity 11%–17%, 23%–29%), *λ* (Sparsity 11%, 12%) and *σ* (Sparsity 13%–17%, 22%–24%) in comparison to HC (blue triangles in Figure 4). After a single session of rTMS, R2 revealed significantly increased *γ* (Sparsity 11%–17%, 20%–24%, 29%–37%), *λ* (Sparsity 11%, 12% and 14%) and *σ* (Sparsity 15%–16%, 22%–24% and 29%–34%, 38%, 40%, 42%–45%) compared to R1 (red quadrangles in Figure 4). The R2 connectivity was not significantly higher than HC.

#### Evidence from rsfMRI

Similarly in rsfMRI also, R1 showed reduced *γ* (Sparsity 21%–40%) and *σ* (Sparsity 25%–34%) in comparison to HC (blue triangles in Figure 4).

After a single session of rTMS, it was found that *γ* increased significantly (Sparsity 16%–43%) in R2 compared to R1. With a decrease in the *λ* (21%–40%) there was a significant increase in *σ* (Sparsity 17%–45%; red quadrangles in Figure 4). The decreased *λ* in R2, compared to HC was found significant at 15%, 16%, 23%–32% and 35%–37% sparsity ranges (black stars in Figure 4). The *γ* and *σ* in R2 compared to HC were not significant.

### Regions Showing Significant rTMS Induced Alterations

The EEG electrodes P4 and FC5 showed significant differences (*p* < 0.05, FDR Corrected) between R1 and R2 groups (Table 1).

**Table 1:** The mean ± SD clustering coefficient (*γ*), Cohen’s standard deviation, the effect size of the regions which showed significant changes after rTMS is presented.

Modalities	Brain region	MNI coordinates	R1 *γ*	R2 *γ*	Cohen’s *d*	Effect size	*p*-value
EEG	P4	(41; −55; 37)	1.81 ± 0.27	2.03 ± 0.21	0.91	0.41	0.004
	FC5	(−56; 1; 21)	1.26 ± 0.31	1.71 ± 0.42	1.22	0.52	0.002
rsfMRI	Right inferior cerebellum	(18; −81; −33)	1.43 ± 0.32	1.69 ± 0.23	0.93	0.42	0.003
	Left anterior insula	(−36; 18; 2)	1.37 ± 0.36	1.63 ± 0.24	0.84	0.39	0.004
	Right medial frontal	(0; 15; 45)	1.42 ± 0.24	1.7 ± 0.34	0.95	0.43	0.005
	Right ventral frontal	(51; 23; 8)	1.31 ± 0.34	1.58 ± 0.22	0.94	0.42	0.001
	Left dorso-lateral prefrontal	(−44; 27; 33)	1.38 ± 0.23	1.73 ± 0.21	1.58	0.62	2.26E-06
	Right Inferior parietal lobule	(54; −44; 43)	1.3 ± 0.28	1.69 ± 0.15	1.73	0.65	6.22E-05
	Left parietal	(−55; −22; 38)	1.26 ± 0.28	1.63 ± 0.19	1.54	0.61	0.001

*The regions and their MNI coordinates are shown with corresponding *p*-values in both EEG and rsfMRI*.

The brain areas which revealed significant differences between R1 and R2 groups on rsfMRI were the right inferior cerebellum, left anterior insula, right medial frontal cortex, right ventral frontal cortex, left dorso-lateral prefrontal cortex, right inferior parietal and left parietal lobes (*p* = < 0.05, FDR Corrected; Table 1). These regions were found to be part of the cerebellar, cingulo-opercular, default mode, fronto-parietal and sensori-motor networks in Dosenbach’s atlas.

### Multi-Modal EEG-fMRI Graph Analysis

**Figure 5:**
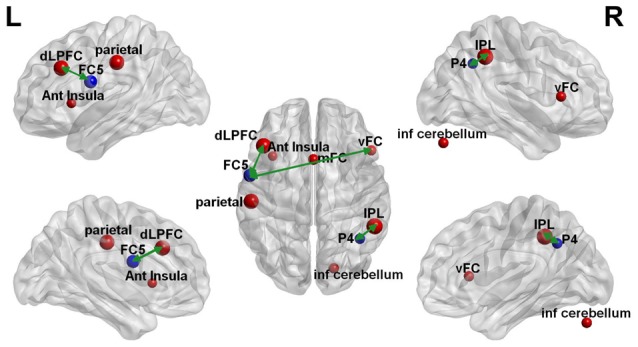
Multi-modal EEG-fMRI graph depicted on a brain surface model. The clustering coefficient of the brain regions that revealed significant changes with rTMS on EEG (blue sphere) and rsfMRI (red sphere) is illustrated proportionate to their effect sizes. The green double edge arrows reveal areas that had significant correlations on EEG and rsfMRI.

The clustering coefficient of EEG electrodes and the rsfMRI brain regions which showed significant changes were correlated using multi-modal graph analysis and plotted on a template (Figure 5). It was found that the clustering coefficient of the P4 electrode was significantly correlated with the right inferior-parietal lobule (IPL) in rsfMRI and the FC5 electrode was correlated to both the right ventral frontal cortex and left dorso-lateral prefrontal cortex (Table 2). The cerebellum, anterior insula, medial frontal cortex and left parietal lobe in rsfMRI did not show any significant correlation in EEG.

**Table 2:** The regions which showed significant (*r* > 0.4) correlation in the multimodal EEG-fMRI graph analysis is presented in the table with the corresponding *r* and *p*-value.

Modalities	Brain region	*γ* Correlation of EEG-fMRI	*r*-value	*p*-value
EEG	P4	Right IPL	0.53	0.04
	FC5	Right ventral frontal cortex;	0.56;	0.03
		Left dorso-lateral prefrontal cortex	0.51	0.05
rsfMRI	Right inferior cerebellum	-	-	-
	Left anterior insula	-	-	-
	Medial frontal cortex	-	-	-
	Right ventral frontal cortex	FC5	0.56	0.03
	Left dorso-lateral prefrontal	FC5	0.51	0.05
	Right Inferior parietal lobule	P4	0.53	0.04
	Left parietal	-	-	-

## Discussion

A single session of low-frequency (1 Hz) rTMS was seen to enhance disease induced loss of small-worldness in several fronto-parietal regions in 14 patients with WC. These changes were consistently seen in both EEG and rsfMRI, probably reflecting acute changes that occur simultaneously at neuronal and hemodynamic networks. The fronto-parietal areas were correlated in EEG-fMRI, while rsfMRI revealed additional areas involving cerebellum, insula, medial frontal and left parietal lobes.

Small-world networks, defined as the ratio of clustering coefficient to pathlength, are considered efficient brain connections. They maximize efficiency with increased cortical clustering at minimum cost due to reduced pathlength (Bassett and Bullmore, 2009). It is well understood that a diseased brain has less efficiency and the networks tend toward either random (Bartolomei et al., 2006; Micheloyannis et al., 2006) or a regular pattern (De Vico Fallani et al., 2007), both reflecting suboptimal brain organization. Many studies have demonstrated alterations in small-worldness in a spectrum of disease conditions like aging (Achard and Bullmore, 2007), stroke (Wang et al., 2010), neuropsychiatric disorders such as schizophrenia and ADHD (Liu et al., 2008; Wang et al., 2009). In the present study, the finding of disease-induced reduction in small-worldness in patients with WC thus concurs with the prior evidence, albeit, in a different disease condition. The focus of the current study is, however, to report that a single session of low-frequency rTMS can restore the disease induced loss of small-worldness. This evidence is in line with several neuroimaging studies consistently revealing an increase in perfusion, task-based activation or resting connectivity after low-frequency rTMS (Havrankova et al., 2010; Eldaief et al., 2011; Bharath et al., 2015). The nature of this acute phase hyperconnectivity was presumed to be either due to increased neuronal coupling between regions or noise due to increased intraregional heterogeneity (Bassett et al., 2011). The usefulness of this hyperconnectivity response was also debated as the information on whether it was coupled with a reduction in long distance connections was less known (Hillary et al., 2015). In the current study (Figure 4), it is interesting to note that the rTMS induced increased clustering coefficient was coupled with reduced pathlength on rsfMRI. In partial contradiction, in EEG, it was observed that rTMS increased the pathlength below 15% sparsity, though above 15% it remained unchanged. The reversal in the direction of group differences across sparsity, could be explained in relation to rTMS-induced changes in a selection of highly dense connections (Phillips et al., 2015), which were not significant enough to surpass the changes in clustering coefficient. It is also possible that this phenomenon is reflective of the methodological differences related to a simple correlation of sparser EEG nodes as this differential response was not observed in rsfMRI. Though it is unclear how definition of edges and nodes would behave while we combine divergent modalities (Yu et al., 2016) it needs to be noted that rTMS increased the small-worldness in both EEG and rsfMRI and there was significant correlation of the EEG-fMRI clustering coefficient in the fronto-parietal regions, coinciding with evidence from prior EEG-TMS studies (Thut and Miniussi, 2009). Hence, based on the concurring evidence from EEG-fMRI, one could presume that rTMS induced hyperconnectivity response is not just a “random noise injected by TMS” as was once assumed (Walsh and Rushworth, 1999; Pascual-Leone et al., 2000). However, further studies, using molecular imaging-TMS, would be required to understand the relationship of this hyperconnectivity to experimentally observed LTD.

Methodologically, there is another study that has explored graph theory analysis of rsfMRI in HCs immediately after a single session of high-frequency rTMS. They found no significant differences in the brain networks after rTMS (Park et al., 2014) probably because HCs have inherent mechanisms to limit significant modifications by a high-frequency stimulation. It is also likely that various technical differences between the studies like smoothing, use of a different parcellation scheme and weighted undirected matrices could have independently modified the results (Wang et al., 2011).

Prior imaging-TMS studies have revealed additional involvement of cerebellum and basal ganglia as partially noted in the current study (Popa et al., 2013; Bharath et al., 2015). These findings were however unexpected in the background of TMS studies revealing region-specific changes in cortical excitability (Gilio et al., 2003; Plewnia et al., 2003) and inhibitory (Gilio et al., 2003; Plewnia et al., 2003) measures after rTMS. Though widespread changes were observed in several TMS-EEG studies (Schutter et al., 2001; Strens et al., 2002; Brignani et al., 2008) it was discredited and was ascribed due to volume conduction issues of EEG (Thut and Miniussi, 2009). However, with the role of higher order fronto-parietal attentional networks in controlling sensory and motor regions (Rushworth et al., 2003) and the evidence of functional connections of cerebellum to prefrontal cortex (Pastor et al., 2006), our finding of widespread changes involving cerebellum, cingulo-opercular, default mode, frontal-parietal and sensori-motor networks assumes greater importance. As the evidence is synchronous with prior imaging-TMS and TMS-EEG studies, one could presume that rTMS induced modulations occur over a range of integrated neuronal assemblies. The continuum of this acute phase response (Battelli et al., 2017) will, however, be required before its clinical relevance to behavior and response to therapy can be ascertained.

As the primary aim of this study was to evaluate the immediate changes induced by rTMS, correlation of these findings with dystonia rating scales was not done to elucidate disease induced network alterations. It intrigued us to see that a single session of rTMS increased the small-world properties over and above the values seen in HC in EEG and rsfMRI, though only decreased pathlength in rsfMRI reached statistical significance. This could represent a methodological error as the HCs did not undergo rTMS, and it is known that rTMS can alter baseline connectivity (Gromann et al., 2012) and perfusion (George et al., 1999) in HCs. Control study using sham rTMS could have been used, but was not performed as it was unplanned at the design stage of the study. More accurate and site specific stimulation could have been achieved using task-based navigation guidance and using an MR compatible rTMS. Spatial concordance of these metrics could have been assessed with high-density EEG and electrode registration with MRI. These techniques were unavailable at our institute during the study. Despite these limitations, the current study has furthered the understanding of brain stimulation on the topology of human brain networks and we believe it will be of particular interest in the understanding of rTMS induced modulations *in vivo*.

## Conclusion

Multi-modal graph theory analysis of EEG and fMRI has concurrently revealed an increased small-worldness in response to a single session of rTMS suggesting that rTMS induced changes are probably not “noise”. Though network alterations in fronto-parietal areas were established on both EEG and rsfMRI, wider involvement of insula, medial frontal brain regions, and cerebellum was found only in rsfMRI.

## Author Contributions

RDB and PKP contributed to the concept and design of the work. Data acquisition was done by MVB and RP, and analysis was carried out by RP, VRR, SB and AP. RDB and SG interpreted the results of the study. Manuscript drafted by RDB, RP, VRR, SB and AP followed by RDB and PKP revised the manuscript critically for important intellectual content. Final approval and agreement to be accountable for all aspects of the work in ensuring that questions related to the accuracy or integrity of any part of the work were appropriately investigated and resolved by all authors.

## Conflict of Interest Statement

The authors declare that the research was conducted in the absence of any commercial or financial relationships that could be construed as a potential conflict of interest. The reviewer JGK and handling Editor declared their shared affiliation.

## Footnotes

^1^www.brain-connectivity-toolbox.net

^2^http://sccn.ucsd.edu/eeglab

^3^http://marsbar.sourceforge.net

^4^http://www.brain-connectivity-toolbox.net
